# ADDRESSING BARRIERS TO A FULL RANGE OF EVIDENCE-BASED OBESITY CARE FOR OLDER ADULTS: A GSA ROUNDTABLE DISCUSSION

**DOI:** 10.1093/geroni/igad104.3162

**Published:** 2023-12-21

**Authors:** John Batsis, Jason Lofton, Anna Pendrey, Kathryn Porter Starr, Jennifer Pettis, Karen Tracy

**Affiliations:** University of North Carolina at Chapel Hill, Chapel Hill, North Carolina, United States; Lofton Family Clinic, De Queen, Arkansas, United States; Indiana University, Indianapolis, Indiana, United States; Duke University School of Medicine, Durham, North Carolina, United States; The Gerontological Society of America, Washington, District of Columbia, United States; The Gerontological Society of America, Washington, District of Columbia, United States

## Abstract

Obesity is identified as a chronic disease by the American Medical Association and several professional societies such as the American College of Cardiology, the American Heart Association, The Obesity Society, and the American Gastroenterological Association. These organizations have all established clinical guidelines on caring for individuals with obesity and overweight. However, there is a critical lack of appropriate and individualized treatment strategies for older adults with obesity. To address this issue, in June 2023 The Gerontological Society of America (GSA) convened 15 multidisciplinary leaders in a roundtable discussion on Addressing Barriers to Accessing a Full Range of Evidence-Based Obesity Care Options by Older Adults. Roundtable participants identified how to overcome obesity/overweight perceptions, communicate appropriately with older adults about their body size, address cultural differences in obesity care, provide care in conjunction with common comorbidities that are faced by older adults, collaborate with an interprofessional team to care for older adults with obesity, overcome barriers to implementation of lifestyle changes including diet and exercise, incorporate medical and surgical interventions, and align needs of older adults with community resources. This poster will illustrate key findings from this roundtable discussion.

